# Prediction of Glioblastoma Multiform Response to Bevacizumab Treatment Using Multi-Parametric MRI

**DOI:** 10.1371/journal.pone.0029945

**Published:** 2012-01-11

**Authors:** Mohammad Najafi, Hamid Soltanian-Zadeh, Kourosh Jafari-Khouzani, Lisa Scarpace, Tom Mikkelsen

**Affiliations:** 1 School of Electrical and Computer Engineering, Control and Intelligent Processing Center of Excellence (CIPCE), University of Tehran, Tehran, Iran; 2 Image Analysis Laboratory, Department of Radiology, Henry Ford Health System, Detroit, Michigan, United States of America; 3 Department of Neurosurgery, Hermelin Brain Tumor Research Center, Henry Ford Health System, Detroit, Michigan, United States of America; Institut Gustave Roussy, France

## Abstract

Glioblastoma multiform (GBM) is a highly malignant brain tumor. Bevacizumab is a recent therapy for stopping tumor growth and even shrinking tumor through inhibition of vascular development (angiogenesis). This paper presents a non-invasive approach based on image analysis of multi-parametric magnetic resonance images (MRI) to predict response of GBM to this treatment. The resulting prediction system has potential to be used by physicians to optimize treatment plans of the GBM patients. The proposed method applies signal decomposition and histogram analysis methods to extract statistical features from Gd-enhanced regions of tumor that quantify its microstructural characteristics. MRI studies of 12 patients at multiple time points before and up to four months after treatment are used in this work. Changes in the Gd-enhancement as well as necrosis and edema after treatment are used to evaluate the response. Leave-one-out cross validation method is applied to evaluate prediction quality of the models. Predictive models developed in this work have large regression coefficients (maximum *R* ^2^ = 0.95) indicating their capability to predict response to therapy.

## Introduction

Brain tumors are considered amongst the most refractory malignancies. Although several therapies have been developed to improve outcomes in such patients, benefits have been relatively modest. Glioblastoma (GBM) is the most malignant type of brain tumor and constitutes about 23% of all primary brain tumors [1]. Multimodal treatment including surgery, radiation, and chemotherapy are used but outcomes remain limited [2], [3].

As tumors need oxygen and substrates for growth, they express growth factors stimulating endothelial cell proliferation and capillary sprouting; or angiogenesis. Vascular Endothelial Growth Factor (VEGF) which initiates the endothelial proliferation is a prime mover in this process. Bevacizumab (Avastin; Genentech, South San Francisco, CA) is a humanized monoclonal antibody which sequesters the ligands VEGF-A and -B inhibiting angiogenesis [4], [5]. Bevacizumab has received accelerated approval from the US FDA for the treatment of refractory GBM.

A problem with current therapies is that their impact on a particular patient may not be known ahead of time, not to mention the significant costs. As such, after several months of treatment, the treatment results may not be satisfactory. A predictive model of the tumor response to treatment is thus very helpful to the physicians and patients as it allows them to select the most effective option.

Mardor, *et al.* [6] addressed this problem using two parameters of diffusion weighted imaging (*ADC* and *R_D_*) in pre-treatment images and showed that these parameters were correlated with the response, defined as relative change in the tumor size. Bezabeh, *et al.* [7] used several parameters of MR spectroscopy, such as elevation of choline resonance, to predict the response of head and neck cancer to radiation therapy. Chen, *et al.* [8] demonstrated that positron emission tomography and the fluorothymidine, as an imaging biomarker, could be used to predict response of malignant glioma to a combination of bevacizumab and irinotecan therapy. Moffat, *et al.* [9] utilized functional diffusion map imaging biomarker and concluded that chemotherapy dose was correlated with this biomarker and the dose itself was also correlated with the response. Lemaire, *et al.* [10] examined tumor treatment in rats and reached the conclusion that there were some relationships between pre-treatment diffusion weighted parameters (e.g., *ADC*) and the tumor size several days after the therapy. Baurle, *et al.* [11] investigated the predictability of the response in patients with breast cancer bone metastasis and showed that the change in the lesion size can be assessed much earlier via the DCE-MRI biomarkers. Swanson, *et al.* [12] developed a model for computing the rate of change in the glioma cell concentration and for estimating the patients' survival, mainly based on two biological factors (net rates of proliferation and diffusion) [13].

Development of a prediction system requires at least two series of images acquired from a number of patients to specify some measure of response. Additionally, using serial images, changes of specific biological and imaging parameters may be traced and their relationship with treatment and time may be investigated. Therefore, many studies have focused on this aspect of medical imaging. There are several sources of error and variance in these images that should be carefully considered in their analysis [14], [15], [16], [17], [18].

The purpose of this work is to establish a relationship between multi-parametric MRI, including T1-weighted pre-Gd (T1-pre), T1-weighted post-Gd (T1-post), T2-weighted (T2), and Fluid attenuated inversion recovery (FLAIR) images acquired pre-treatment, and the reduction in the Gd-enhanced volume due to bevacizumab treatment. The differences among the Gd-enhanced regions of different patients in terms of their homogeneity and brightness has motivated us to extract their characteristics and features to stratify responders from non-responders and develop a predictive model for the level of response. In addition, analysis of the data acquired from the patients in several consecutive imaging series (including the pre-treatment session) is performed to see how the patients' conditions are affected by the therapy and how the tumor characteristics are influenced during the treatment time interval. To the best of our knowledge, this work is the first study that uses multi-parametric structural MRI to predict the response to therapy.

## Materials and Methods

### Ethics Statement

This research has been approved by the Henry Ford Health System Institutional Review Board. We obtained written informed consent from all participants in the study.

Twelve patients (9 males, 3 females; age range 36–66, mean 54) with GBM and Gd-enhanced areas in their T1-post were chosen for the study. All of the patients had edema encompassing the tumors and 8 patients had necrosis. Tables 1 and 2 present tumor locations, treatments delivered, imaging characteristics, age, and gender of the patients.

**Table 1:** Summary of patients, locations of tumors, and treatments delivered at different dates.

Patient ID	Tumor Location	Date of Diagnosis	First Progression Date Recorded	Last Progression Date Recorded	Treatments Delivered at Different Dates
1670	L Frontal	8/29/2006	10/3/2006	9/27/2007	EBRT+TMZ, TMZ, Iri+Bev, Bev+Tar
178	R Frontal	1/25/2002	9/9/2002	12/17/2008	EBRT, CCNU, Pro, Iri, TMZ, Iri, Bev, EM1421
1125	Multiple	3/17/2006	9/28/2006	3/13/2008	EBRT+TMZ+Tal, TMZ, Bev
1847	L Temporal	6/19/2006	10/5/2006	5/23/2007	EBRT+TMZ, TMZ, Iri+Bev
1197	L Frontal	4/1/1994	2/1/1999	11/9/2010	EBRT, BCNU, PCV, SRS, TMZ, Iri, Iri+Bev, TMZ+Bev
1170	R Parietal	8/28/2003	1/21/2005	3/31/2009	EBRT+TMZ, TMZ, SRS, TMZ, CPT11+Bev
972	R Frontal	12/23/2005	7/1/2006	5/5/2008	EBRT+TMZ, TMZ, Iri+Bev, TMZ
969	L Occipital	12/19/2005	8/10/2006	10/5/2010	EBRT+TMZ, TMZ, Iri+Bev, AT-101, TMZ
102	L Temporal	8/7/2000	1/20/2003	7/5/2006	EBRT, TMZ, Bev, Bev+Iri, Bev+Car
852	L Temporal	10/27/2005	5/18/2006	1/23/2007	EBRT+TMZ, TMZ, Iri, Bev
1876	R Temporal	1/22/2005	4/2/2007	-	EBRT+TMZ, TMZ, Iri+Bev
1589	R Temporal	8/14/2006	4/24/2007	11/29/2007	EBRT+TMZ+Cil, TMZ, MLN-518, TMZ+Bev

The following abbreviations are used: External Beam Radiation Therapy (EBRT), Temozolomide (TMZ), Bevacizumab (Bev), Irinotecan (Iri), Caroplatin (Car), Procarbazine (Pro), Talamanel (Tal), Ciligentide (Cil), Tarceva (Tar), Lomustine (CCNU), Carmustine (BCNU), Stereotactic Radio-Surgery (SRS), Procarbazine, CCNU and Vincristine (PCV), targeted chemotherapy agents (MLN-518, AT-101), irinotecan aka Camptosar (CPT11), targeted chemotherapy agent (EM1421).

**Table 2:** Summary of the imaging characteristics of the patients along with age and gender information.

Patient ID	Gd-enhancement	Necrosis	Age	Gender
1670	Irregular	Inside Gd-enhancement	62	M
178	Irregular	No	36	M
1125	Round	Inside Gd-enhancement	66	F
1847	Irregular	Inside Gd-enhancement	62	M
1197	Small but scattered	Very sparse	49	M
1170	one round, one irregular	Two regions, one inside Gd	47	M
972	Round, inside gray matter	No	56	F
969	Irregular	Not adjacent with Gd-enhancement	60	M
102	Irregular, scattered	No	52	M
852	Round and Irregular	Inside Gd-enhancement	57	M
1876	Two round and irregular foci	Two regions, both inside Gd	41	F
1589	Round and irregular	No	55	M

All patients had edema.

Several series of MR images were acquired for the patients, once before starting the treatment and then with the time intervals of 1–3 months after the treatment (Henry Ford Health System, Detroit, MI, USA). These images were acquired using a 3 T GE system and included multi-parametric images with an image matrix size of 512×512: T1-weighted with TR/TE/TI = 3000/6/1238 ms, T1-post with TR/TE/TI = 3000/6/1238 ms, T2-weighted with TR/TE = 3000/103 ms, and FLAIR with TR/TE/TI = 10000/120/2250 ms. The images had high quality and were already co-registered, so no noise reduction or registration step was applied. The patients had different number of image acquisitions. For example, whereas five patients had four series of image acquisitions, four patients experienced three series, and three patients had two series of images. Because of this non-uniformity, the prediction of the response to therapy was done based on the second image series. Time intervals between the first two series of images of the patients range from 41 days to 83 days.

First, all slices in the T1-post images of each patient were examined to select the ones with Gd-enhanced areas for volume analysis. Then, the skull was removed in the selected slices using Eigentool (http://www.radiologyresearch.org/eigentool.htm). Next, Gram-Schmidt orthogonalization was applied to the baseline MR images. As explained below, this approach decomposes the multi-parametric MRI data into white matter (WM), gray matter (GM), cerebrospinal fluid (CSF), and the remainder (orthogonal) composite images [19]. Imaging features are later extracted from these composite images. The application of Gram-Schmidt orthogonalization makes the analysis independent of the intra- and inter-patient variations in intensity and contrast of the MR images, thus resulting in features robust to such variations.

Initially, some samples from the pixels of each region to be segmented (i.e., WM, GM, and CSF) are manually chosen. These pixels are considered as the desired tissue pattern and the pixels from the other regions are regarded as undesired tissue patterns. Each composite image is constructed using a weighting vector that projects the original multi-dimensional vectors defined using the original MR images to a specific subspace:
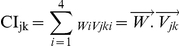
where 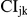 is the intensity of the 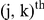 pixel in the composite image, 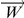 is the weighting vector, and 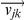 is the intensity vector of the 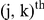 pixel in the original MR images. To find the weighting vector of each composite image, its SNR is maximized while the inner products of the weighting vector with the other tissue patterns are zero [20]. Under these conditions, the resultant weighting vector is:
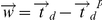
where 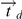 is the desired tissue vector and 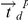 is the projection of 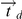 onto the undesired tissue vectors (subspace). The latter can be calculated using the Gram-Schmidt orthogonalization procedure [21].

**Figure 1:**
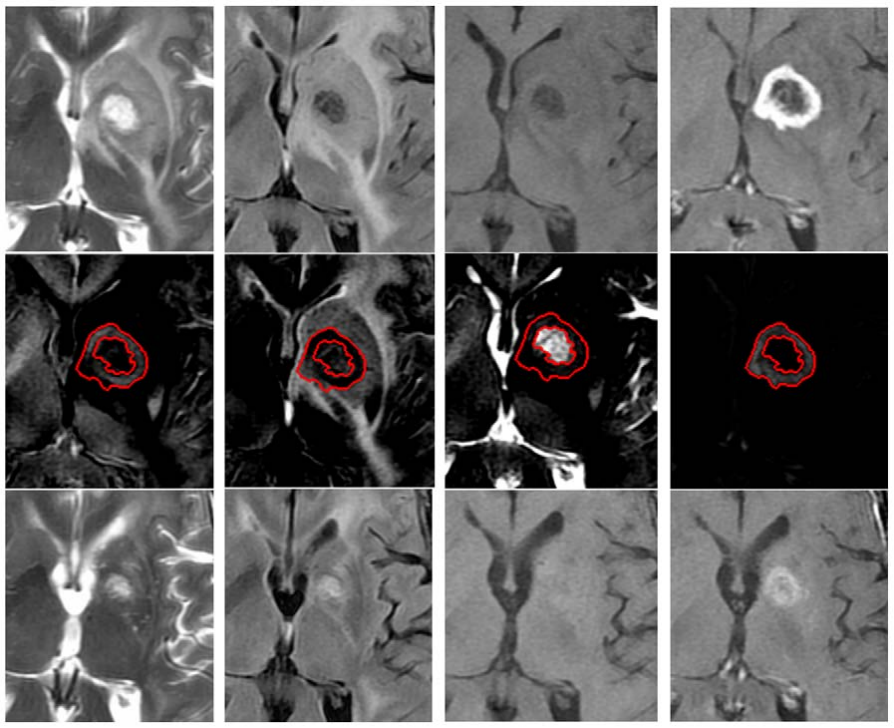
An FOV from multi-parametric MRI and the resulting composite images of a responder. (Patient ID: 1125), 1st row: MR images before the treatment (T2, FLAIR, T1-pre and T1-post, respectively from left to right). 2nd row: Composite images (WM, GM, CSF and Orthogonal, respectively from left to right). 3rd row: MR images acquired 41 days after the treatment. Red ROIs show borders of Gd-enhanced region on different images.

**Figure 2:**
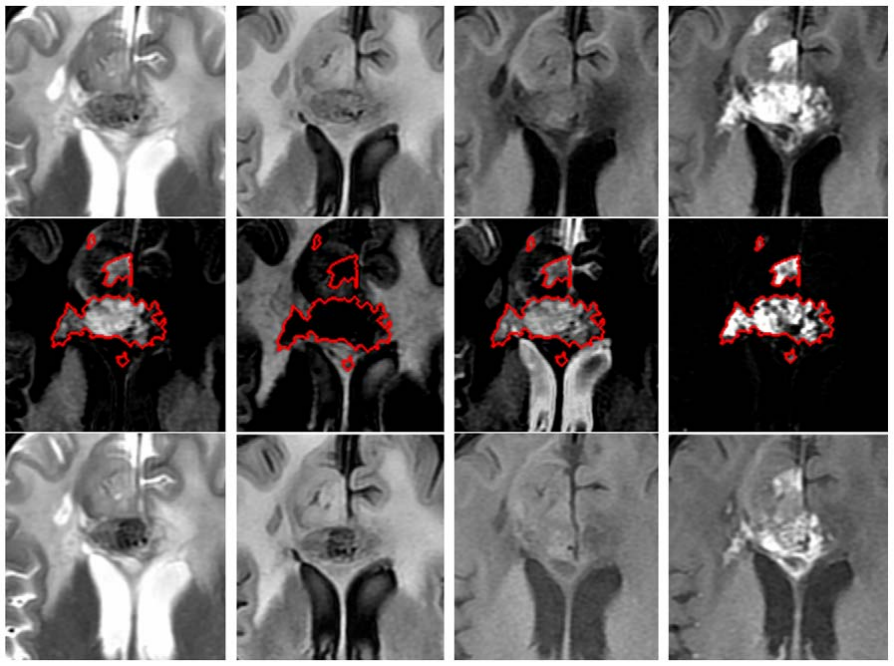
An FOV from multi-parametric MRI and the resulting composite images of a non-responder. (Patient ID: 178), 1st row: MR images before the treatment (T2-weighted, FLAIR, T1-weighted and T1-post, respectively from left to right). 2nd row: Composite images (WM, GM, CSF and Orthogonal feature, respectively from left to right). 3rd row: MR images, acquired 50 days after the treatment. Red ROIs show borders of Gd-enhanced region on different images.

The above step was also implemented and applied in Eigentool. In this approach, different regions of the affected area are distributed into different composite images. For example, the Gd-enhanced area of the tumor appears in the WM, CSF, and orthogonal images but not in the GM image. Also, the edema is mostly projected onto the GM image. Note that the projection is based on MR image intensity, not physical location of the tissues. Edema is more similar to GM than to WM in the images. Figures 1 and 2 show original and composite images for sample responder and non-responder patients (as defined later), respectively. Note that the enhanced area and edema of the responder have decreased. Also, note that the Gd-enhanced region is clearly visualized (segmented) in the orthogonal images.

To define the Gd-enhanced area, the T1-post image was divided by the T1-weighted image pixel by pixel and the result theresholded. This method requires that the two images have similar brightness. To this end, a normalization step was applied to the images by selecting an ROI in the unaffected WM on the T1-pre image and its corresponding region in the T1-post image. Then, the average intensities of the pixels in this region of the two images were calculated and the relative gain of the two images was obtained by dividing their average intensities. The gain was used for the normalization of the images.

The process of ROI definition was performed for the edema and necrosis as well. For this aim, a simple thresholding was applied to the FLAIR and T1-post images to extract edema and necrosis, respectively. To treat all of the ROIs equally, an identical threshold should be used for all of the images from the same modality. Therefore, the adverse effect of the intensity gain in some images (especially in FLAIR images) was eliminated by normalization of the intensities. For example, to extract the ROI of edema, the edema in a sample FLAIR slice was first segmented manually. Then, the average intensity of this region was computed and divided by the average intensity of an arbitrary ROI in the WM region of that image. This ratio was used to find the threshold value in all other FLAIR slices and define volumetric ROI of the edema. Although this method may not be very accurate, it is sufficient for our study because histogram features are utilized that consider the pixels in the ROI as an aggregate and thus a few pixel outliers do not affect the resulting features.

Using the ROI of the Gd-enhancement and the thickness of each slice, the volume of the Gd-enhanced area was computed. This process was repeated for all of the image series. Then, the relative change in the volume of the Gd-enhancement between the baseline and second image series was recorded as a measure of response. This is due to the limitation that only two images series were acquired for some of the patients.

**Figure 3:**
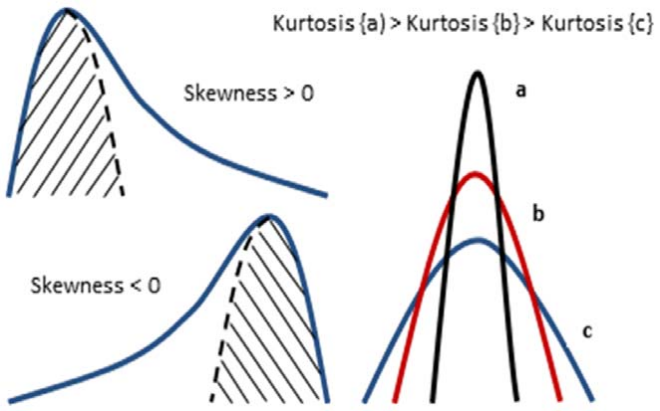
Interpreting the properties of skewness and kurtosis in histograms.

Next, a central slice of each volume was chosen for statistical feature extraction (tissue characterization). It should be noted that the tissue characteristics can be reliably measured in the areas without considerable partial volume effects. The central slice has the minimum amount of partial volume and thus can yield the most accurate tissue features [20]. In this step, ROI of the Gd-enhanced area was overlaid onto the composite images (WM, GM, and CSF) and their histograms were calculated. Then, a normalization step was applied to them to compensate for the effect of the ROI size. Four histogram features (Mean, standard deviation, skewness, and kurtosis) were extracted. Mean and standard deviation, represent average and dispersion of the histogram, respectively. Skewness is a measure of the histogram asymmetry and kurtosis reflects sharpness of the histogram peak [22]. The properties of the last two parameters are illustrated in Figure 3. Altogether, 12 features were extracted from the three composite images. Note that the features are extracted from baseline MR images, whereas the response is measured by comparing the baseline and second series of MR images.

We established one-dimensional and multi-dimensional relationships between the proposed features and the extent of response in patients. To this end, single and multiple-regression analyses were done on the results. Prediction equations and the corresponding regression coefficients were derived from these analyses. To control the false discovery rate, we adopted the multiple testing algorithm proposed in [23]. In addition, leave-one-out cross validation was performed on the data to evaluate the predicted results based on the actual responses of the patients. Also, changes in the volumes of edema and necrosis were evaluated to investigate if they had any relationship with the response of the brain tumor to treatment.

Besides these statistical features, we also analyzed the shape and size of necrotic areas of the tumors to see if there were any dependencies between these parameters and the amount of response to the therapy in the patients. This region was selected for this analysis due to its impact on the tumor growth or treatment (It will be discussed in conclusion).

## Results

**Figure 4:**
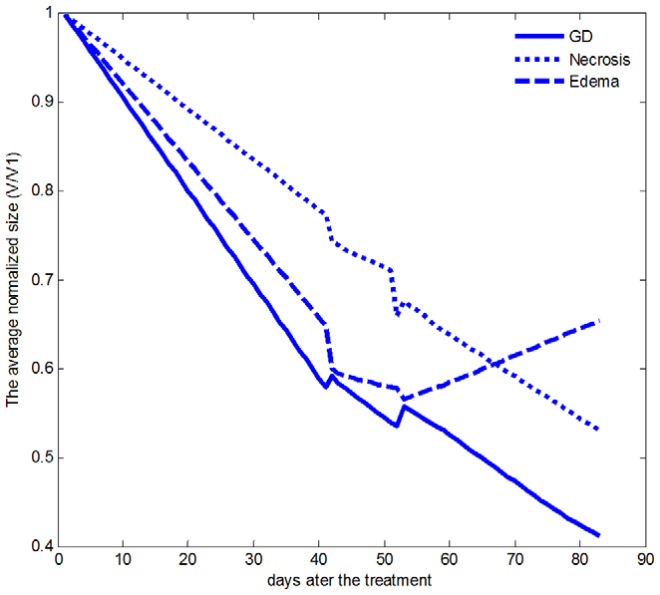
Averages of normalized volumes of Gd-enhancement, edema, and necrosis for all patients after treatment.

The volumes of Gd-enhancement, edema, and necrosis were estimated for all patients at different acquisition times. The volume of each region was normalized to its baseline volume. Then, an overall curve of size versus time was obtained for each region by averaging the values estimated for all patients. Figure 4 illustrates the average curves for the Gd-enhanced area, edema, and necrosis, up to 83 days after the treatment. On average, Gd-enhanced and Necrosis regions decreased in size whereas edema initially decreased but then increased.

All patients experienced a decrease in the Gd-enhanced region. Table 3 reports the relative volume change of the Gd-enhanced area for the second series of images computed as (*V*_1_−*V*_2_)/*V*_1_, along with the time between the first two image acquisitions and the survival days. This relative change was chosen as a measure of response because it is a normalized version of what is considered a clinical measure of response.

**Table 3:** Tumor volumes and treatment effects on the tumors and time lengths between acquisitions as well as survival length.

Patient ID	Tumor volume in CC (Gd-enhancement+Edema+Necrosis)	Relative change in the volume of Gd-enhancement (%)	Edema relative change (%)	Necrosis relative change (%)	Time between the two image acquisitions (days)	Survival after treatment (days)
1670	89.21	54	47	49	58	225
178^*^	129.90	32	32	-	50	259
1125	56.16	69	51	66	41	336
1847	176.49	67	40	50	41	210
1197^*^	131.67	39	10	−3	54	275
1170	53.37	72	40	46	43	337
972	33.05	83	83	-	42	343
969^*^	18.73	23	−20	−46	52	390
102^*^	100.48	78	0	-	75	396
852	120.64	67	51	5	40	209
1876	252.85	75	85	46	52	863
1589	70.83	55	93	-	83	528

Tumor volumes before bevacizumab treatment as well as relative changes in the Gd-enhancement, edema, and necrosis, between baseline MRI and the one acquired about 2–3 months after the treatment, calculated by (*V*_1_−*V*_2_)/*V*_1_, length of time between two image acquisitions, and survival times of the patients.

(*:tumors without necrosis or those with minimal adjacency of Gd-enhanced and necrotic areas).

**Figure 5:**
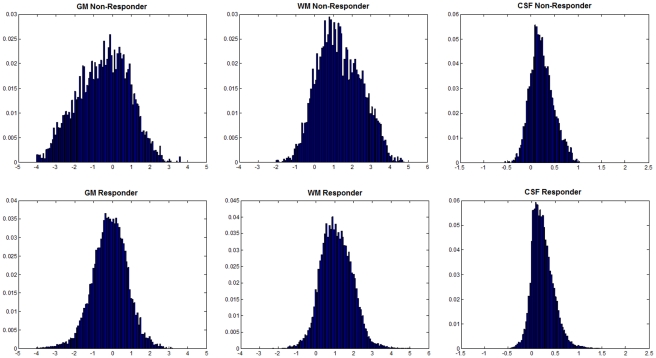
Comparison of image histogram in different tissues in responders and non-responders. Average histograms of GM image (left), WM image (middle), and CSF image (right) for non-responders (top row) and responders (bottom row).

Next, the histograms of the Gd-enhanced region of each patient in the composite images were generated. Figure 5 demonstrates the average histograms of the resultant composite images for the responders (response >50%) and non-responders (response <50%). A significant difference is observed in the shapes of the histograms between the responders and the non-responders, in particular for the GM and WM composite images. This suggests that it might be possible to predict the response to therapy using the histogram statistics. Therefore, the four features described in the previous section were extracted from the histograms of WM, GM, and CSF composite images resulting in 12 features. The central slice of the tumor was selected to minimize the partial volume averaging effects.

**Figure 6:**
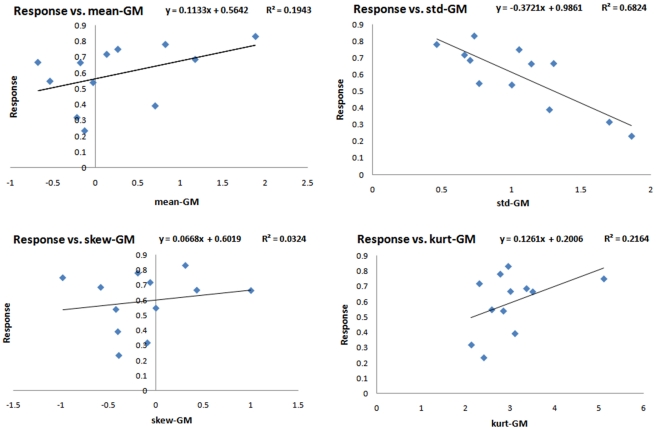
Response versus features extracted from the GM composite image.

**Figure 7:**
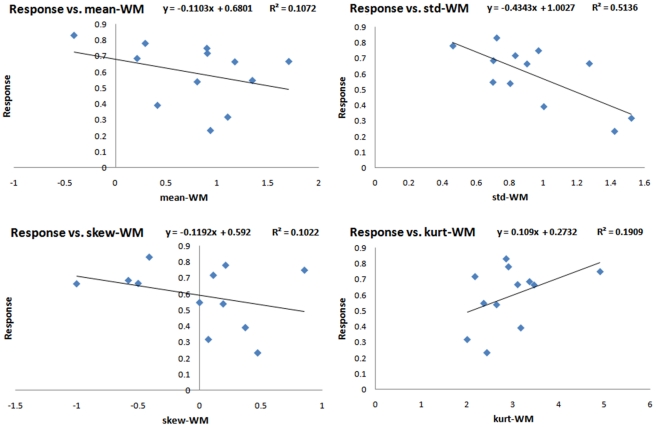
Response versus features extracted from the WM composite image.

**Figure 8:**
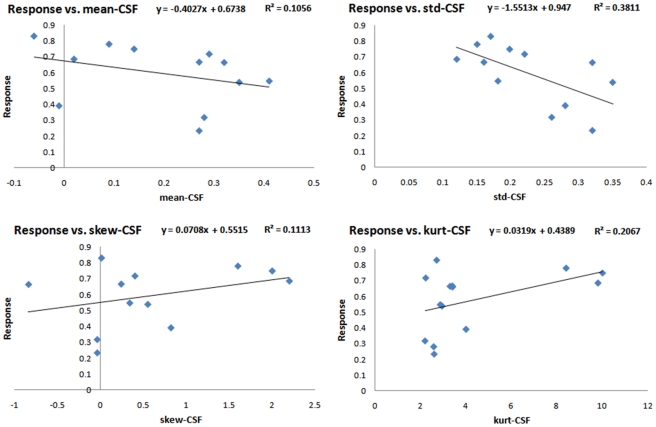
Response versus features extracted from the CSF composite image.

Linear single-regression analysis was performed to develop a model for estimation of the response to therapy using individual features. The features with highest correlation with the response to therapy are presented in Table 4. As we are testing 12 features individually to evaluate how well they are correlated with the response to therapy by reporting their *p*-values, we need to make corrections in this multiple testing experiment to avoid false discovery. To control the false discovery rate, we adopted the algorithm proposed in [23]. Considering 12 hypotheses corresponding to the 12 features, we tested all the *p*-values [24] and found GM-std and WM-std significantly correlated to the response to therapy. The standard-deviation of the GM composite image was, in particular, the best predictor of the response with the highest regression coefficient (*p*<0.0009, *R* = −0.83). Figures 6, 7 and 8 show the plots of response versus individual features extracted from the GM, WM and CSF composite images, respectively. Regression lines and prediction equations are presented for all cases.

**Table 4:** The three features most significantly correlated with the response to therapy.

Feature	Regression coefficient (*R* ^2^)	Correlation coefficient with the response (*R*)	*p*-value
GM-std	0.68	−0.83	*p*<0.0009
WM-std	0.51	−0.72	*p*<0.009
CSF-std	0.38	−0.62	*p*<0.03

To improve the prediction, linear multiple-regression was also applied to the data which improved the regression coefficient (Table 5). A maximum regression coefficient of 0.95 (*Significance F* = 0.0008, *R^2^* = 0.95) was achieved which is superior to the single-regression with the maximum regression coefficient of 0.68. The candidate variables for this analysis were chosen from the ones that had the highest single regression coefficients because each of them could estimate the response quite well. Actually multiple regression analysis was expected to give us a significantly higher regression coefficient because nearly most of the extracted features were almost uncorrelated (Table 6). It should be noted that in the multiple-regression, the measure of “*Significance F*” is used to determine the statistical significance of the results [25].

**Table 5:** Multiple regression results for the prediction of the response to therapy using imaging features.

Features	Regression coefficient (*R* ^2^)	Significance *F*
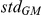 + 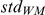	0.73	0.003
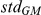 + 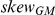 + 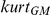	0.87	0.0007
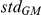 + 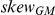 + 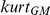 + 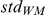 + 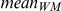	0.95	0.0008

**Table 6:** Correlation coefficients between the features used in the multiple regression analysis.

Features	Correlation coefficient
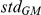 vs. 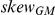	0.001
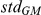 vs. 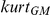	0.15
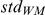 vs. 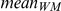	0.51
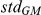 vs. 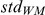	0.95

Survival length was considered as another measure of the response to therapy [26]. The median progression free survival of the patients was 336 days. However, no significant correlation between this measure and the extracted features was found (Significance *F*<0.19).

By comparing Tables 2 and 3, it can be seen among the patients who had necrosis (8 patients), in 6 of them, the response was higher than 50%. This motivated us to look for relationships between size or shape of necrotic area and response to therapy. Although no dependency was found between the two, we observed that the tumors in which the necrotic area was inside the Gd-enhanced area and the tumors that had the largest interface between these two areas had highest levels of response (Tables 2 and 3).

Next, we analyzed the correlation between the relative changes in the Gd-enhanced area (response), edema, and necrosis (Table 3). There was a high correlation (*p<0.006, R = 0.83*) between the Gd-enhanced area relative change (response) and the necrotic area relative change in the patients with necrosis. There was no significant correlation (*p<0.23, R = 0.35*) between the relative changes in edema and the response to therapy. However, a high and significant correlation coefficient (*p<0.0007, R = 0.91*) between these two markers was achieved for the tumors with necrosis. Besides, we found a strong correlation between relative changes in edema and necrosis (*p<0.02, R = 0.8*).

**Figure 9:**
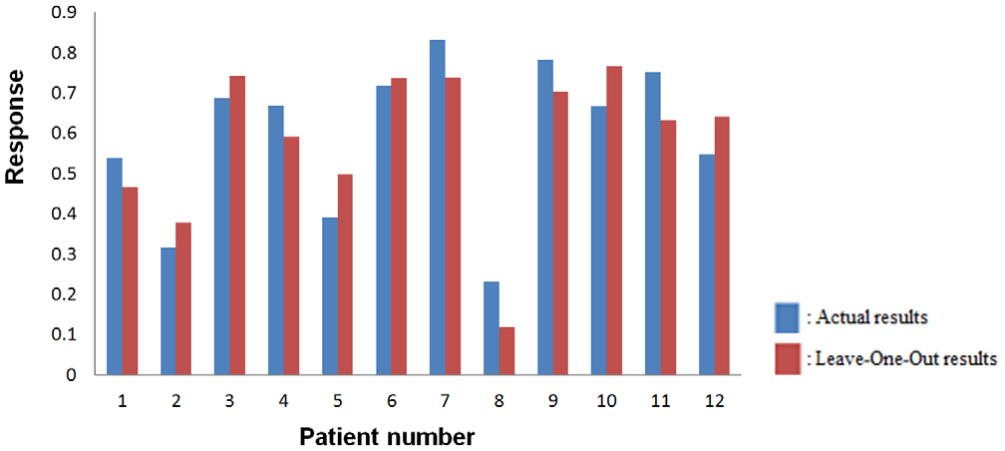
Comparison of the actual responses and estimates responses using Leave-One-Out method. Responses estimated using leave-one-out and the five features in the last row of Table 5 (red columns) compared to actual results (blue columns). The relative change in the size of the Gd-enhanced region was used as the measure of response. The horizontal axis shows the patient that has been eliminated in leave-one-out analysis.

Finally, we performed a leave-one-out cross validation analysis to verify the goodness of fit for the linear model. To this end, using relative change in the size of the Gd-enhanced region as the measure of response and the five features in the last row of Table 5, we performed linear multiple-regression analysis 12 times with eliminating one patient at a time. Then, using the generated linear model, we estimated the response for the remaining patient. Figure 9 compares the estimated and actual values for the 12 patients.

## Discussion

In this study, patients with GBM and Gd-enhanced areas were studied to establish a correlation between the response to bevacizumab treatment and features extracted from the structural MR images. Since the Gd-enhanced area of the tumor reflects the most active region of the tumor, the relative change in the volume of this region was considered as a measure of response. The enhancement of this region in the post-contrast images is mainly due to the leaky capillaries and vessels in the tumor area that allow passage of the contrast agent into the inter-cellular space as a result of their damaged blood brain barrier. The angiogenesis process that facilitates tumor growth makes new vessels weak and highly permeable [27]. Anti-angiogenic therapies control the development of new capillaries and as a result control and even reduce the size of Gd-enhanced area [27]. Therefore, the change in the volume of the Gd-enhanced region reflects the impact of anti-angiogenic treatment on the patients and was evaluated in this work.

Gram-Schmidt orthogonalization analysis was used as it generates more robust features compared with the conventional methods of MRI feature extraction [28]. In this analysis, the gray levels of the desired tissue in the composite images are always distributed around unity and thus, regardless of the intensities of the original images, normalization is not needed.

To develop predictive models of response, single-regression was used to test the correlation between the extracted features and the response to therapy within 1–3 months post-treatment. We used linear regression model which is a model with the minimum number of parameters and potentially highest generalization. Although non-linear models are able to better fit the data, they need a larger number of samples to estimate the model parameters and may have relatively poor generalization. The resultant regression coefficients showed that the linear model was appropriate for our goal.

Relative change in the Gd-enhanced volume was chosen as a measure of response because it provides a more accurate tumor assessment compared with the other methods such as 1D or 2D or even 3D measurements where volume assessment is based on the major diagonal diameters of the tumor [29], [30].

The standard deviation of the GM histogram was found to be the most significant feature for the prediction of the response to therapy. This was to some extent predictable because the standard deviation of the histogram of a specific ROI represents the heterogeneity of the corresponding cancerous tissue and the more a tumor is heterogeneous, the more dangerous and fatal it is which means there is less chance for being able to treat the tumor [31], [32].

Multiple-regression was also performed to attain a more accurate prediction relative to the single-regression analysis. This is due to the fact that each of the variables used for the regression was predictive of the response and most of them were almost uncorrelated (Table 6). On the other hand, the GM-std and WM-std were found highly correlated (Table 6). That is why combining WM-std and GM-std increased the regression coefficient by only 0.02. This is consistent with a finding in [6] where two features (ADC and a diffusion index named RD) were used for prediction. Although both features predicted the response with a good correlation (*R* = 0.76 and 0.77, respectively), they were highly correlated (*R* = 0.95) and thus multiple-regression analysis did not improve the prediction accuracy.

**Figure 10:**
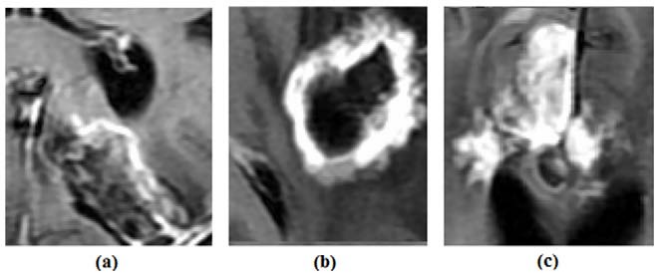
Three different cases of neighborhood between Gd-enhanced area and necrosis. a) Gd-enhanced area is not fully adjacent to necrosis (Patient ID: 969). b) Gd-enhanced area contains necrosis and there is complete adjacency (Patient ID: 852). c) There is no necrosis (Patient ID: 178).

We found that the tumors with necrosis adjacent to the Gd-enhanced areas were more likely to respond to treatment relative to the other tumors. This may be due to the fact that the cells surrounding the necrotic areas are influenced by hypoxia which makes them express the highest amount of VEGF among the tumor cells [33], [34]. This leads us to believe that angiogenesis may be the main mechanism behind the growth of these tumors. Consequently, bevacizumab (anti-angiogenic therapy) is probably the best treatment in such cases. In addition, we noted that bevacizumab has favorably influenced the tumors without necrosis but this influence is not as strong. This result is in concordance with the findings of [35]. Figure 10 displays GBM tumors in 3 patients where in (a), the Gd-enhanced area is not fully-adjacent to necrosis, in (b) there is a maximum of adjacency between these two regions and in (c) the tumor lacks necrosis.

It seems that bevacizumab has not had a positive impact on the edema in the long term (Figure 4). One reason may be the fact that angiogenesis is not the only effective factor for the edema growth [36], [37]. However, it can be seen in Table 3 that the four lowest levels of decrease in the edema size between the first two image acquisitions happened for the tumors without necrosis or those with minimal adjacency of Gd-enhanced and necrotic areas (starred in the table). These results suggest that there is unlikely that angiogenesis would be the main factor for tumor infiltration and development in these cases. This may be one potential explanation to why an anti-angiogenic therapy has not worked well for these cases. It should be also noted that these relative changes are just for second series of the images and many of the patients had an increase in their edema size after a while as it can be seen in Figure 4.

Analysis of the necrotic area was also performed in this study. Figure 4 reveals that the average normalized size of this region in patients with necrosis, at the end of the treatment trial, has considerably decreased. This is in contradiction with a statement in [38] suggesting that the change in the necrotic area would only be possible through surgery.

Leave-one-out cross validation analysis was performed to compare predicted and actual responses of the patients. For some patients, the predicted response was not very close to the actual responses (Figure 10). This is due to the fact that the regression line calculated using the robust linear regression analysis does not pass through the actual results.

Altogether, most of the patients have shown a relatively good level of response to bevacizumab. However, no relationship between this measure and patient survival length was found (Table 3) (*p<0.7, R = 0.13*). One reason may be that the anti-angiogenesis drugs normalize the vascularity in the tumor area and repair the blood brain barrier in this region without any specific anti-tumor effects. This may be an explanation as to why bevacizumab suppresses the Gd-enhanced area but has no significant effect on the non-enhanced areas of the tumor [39], [40]. Since the survival of the patients does not only depend on the Gd-enhanced area of the tumor, no specific relationship between the relative change in the size of this region and survival was found.

A practical limitation of this study is that the time between the first two image acquisitions is not always the same, which may have deteriorated the regression analysis results. Yet, this study illustrates that it is possible to predict response of a brain tumor to bevacizumab treatment before the treatment starts. Such a prediction system may be instrumental for physician selection of optimal treatment for their cancer patients.

In future studies, we intend to extract and evaluate other imaging features from the Gd-enhanced and other sub-regions of the tumor. We also intend to extend the proposed approach to other tumor types and treatment options. Furthermore, we may model shrinkage of the tumor cells based on the shape and texture features of specific image regions.
